# Monitoring the wild black bear's reaction to human and environmental stressors

**DOI:** 10.1186/1472-6793-11-13

**Published:** 2011-08-17

**Authors:** Timothy G Laske, David L Garshelis, Paul A Iaizzo

**Affiliations:** 1 Cardiac Rhythm Disease Management, Medtronic, Incorporated, 8200 Coral Sea Street NE, MVS46, Mounds View, MN, USA 55112; 2 Minnesota Department of Natural Resources, 1201 E Hwy 2, Grand Rapids, MN, USA 55744; 3 Department of Surgery, University of Minnesota, B172 Mayo, MMC 195420 Delaware Street SE, Minneapolis, MN, USA 55455

**Keywords:** Electrophysiology, Hibernation, Cardiac Physiology

## Abstract

**Background:**

Bears are among the most physiologically remarkable mammals. They spend half their life in an active state and the other half in a state of dormancy without food or water, and without urinating, defecating, or physical activity, yet can rouse and defend themselves when disturbed. Although important data have been obtained in both captive and wild bears, long-term physiological monitoring of bears has not been possible until the recent advancement of implantable devices.

**Results:**

Insertable cardiac monitors that were developed for use in human heart patients (Reveal® XT, Medtronic, Inc) were implanted in 15 hibernating bears. Data were recovered from 8, including 2 that were legally shot by hunters. Devices recorded low heart rates (pauses of over 14 seconds) and low respiration rates (1.5 breaths/min) during hibernation, dramatic respiratory sinus arrhythmias in the fall and winter months, and elevated heart rates in summer (up to 214 beats/min (bpm)) and during interactions with hunters (exceeding 250 bpm). The devices documented the first and last day of denning, a period of quiescence in two parturient females after birthing, and extraordinary variation in the amount of activity/day, ranging from 0 (winter) to 1084 minutes (summer). Data showed a transition toward greater nocturnal activity in the fall, preceding hibernation. The data-loggers also provided evidence of the physiological and behavioral responses of bears to our den visits to retrieve the data.

**Conclusions:**

Annual variations in heart rate and activity have been documented for the first time in wild black bears. This technique has broad applications to wildlife management and physiological research, enabling the impact of environmental stressors from humans, changing seasons, climate change, social interactions and predation to be directly monitored over multiple years.

## Background

Data loggers (archival tags) that are attached or surgically implanted in animals to collect and store or relay information about activity, movements, physiology, and the local environment are becoming increasingly sophisticated and useful to biologists [1,2]. Such devices have been used to monitor heart rate and activity of free-ranging reptiles, birds, and mammals [3-5]. With rapid advancements in electronics over the past decade, data logger packages have become smaller and less invasive while the period of monitoring has increased from days to months, and rarely up to 1 year [6,7]. With the aid of these devices, biologists have been able to study critical changes in animal physiology related to their natural history (e.g., migration, foraging dives, fasting) or in response to human disturbance [8-11].

One of the most profound changes in physiology is hibernation, and one of the most physiologically remarkable hibernators is the bear. Smaller hibernators have bouts of arousal and survive the winter in secluded burrows where risks of predation are minimal. By contrast, bears pass the winter in a state of shallow hypothermia without bouts of active arousal [12,13]. However, because of their large mass, they are often in partially-exposed dens with associated higher risks for predation and/or external disturbance [14,15]. Although they have depressed metabolic functions during this period, we have commonly noted defensive posturing and high respiratory rates by bears within a very short period (seconds) of being disturbed. Bears have limited loss of skeletal muscle protein and strength during winter [16-18]. Likewise, their heart is able to revert from the quiescent state of hibernation, conserving energy for up to 6 months of fasting, to supporting a burst of activity in a very short interval. In this respect, the black bear's heart function may be unique among mammals.

Earlier studies reported significant reductions in bears' heart rates, contractility, mass, and output when comparing summer and winter [19-21]. Additional work indicated that cardiac wall thickness and function (electrophysiological parameters) were maintained during the period of hibernation [22]. We previously observed winter heart rates as low as 4.5 bpm, with a dramatic respiratory sinus arrhythmia (RSA) enabling the heart to rest between inspirations [22]. We hypothesized that the RSA is an adaptive mechanism to conserve energy while maintaining adequate cardiac perfusion over winter to sustain the "fight or flight" response.

Previous findings about the seasonal adaptations of ursid hearts were based upon data obtained at a limited number of discrete time intervals from animals that were either chemically anesthetized or hand-reared and trained for these procedures [19-22]. Recent advances in implantable devices applied to the management of human clinical patients have the potential to remove the current barriers associated with long-term monitoring in the wild. In this study we sought to document for the first time annual trends in heart rate and activity from continuous monitoring of free-ranging bears. By studying undisturbed bears in the wild, we sought to elucidate both the physical and environmental situations (seasonal changes, entering/emerging from hibernation, changes in the availability of food, birthing of cubs) and mechanisms (interaction of heart rate, respiration rate, and activity) that motivate their physiological and behavioral changes.

## Methods

Wild radiocollared bears in northern Minnesota (N = 14) were located in their winter dens, anesthetized (Telazol^®^, veterinary formulation of tiletamine and zolazepam) and temporarily extricated. (See Additional Files 1, 2, 3 and 4 for video sequences of hibernating bears during den visits.) Ethylene Oxide sterilized Insertable Cardiac Monitors (ICMs) that were developed for human heart patients (Reveal^® ^XT, Model 9529; Medtronic Inc., Minneapolis, MN; 9 cc; 8 mm × 19 mm × 62 mm; 15 grams) were surgically implanted in these bears in the field using aseptic techniques. An additional device was implanted in a wild orphaned cub that denned in an outdoor enclosure at a rehabilitation center and was released into the wild the following spring. Devices were placed subcutaneously in a peristernal location [23]. The device electronics are housed in a hermetically sealed titanium can. Electrocardiograms are recorded from a differential voltage measured between a titanium electrode housed in a polyurethane and silicone header with a region on the parylene coated titanium can serving as the reference electrode. This device became available to clinicians in the United States in 2009 and has two electrodes on the body of the device to continuously monitor the subcutaneous electrocardiogram (ECG). A built-in accelerometer measures patient activity. Device programming and data retrieval is non-invasive via transcutaneous telemetry associated with a programming system (CareLink Model 2090 Programmer with software Model SW007, Medtronic Inc., Minneapolis, MN).

In addition to storing the timing of each heartbeat and daily activity over the three year life of the device, the device memory can store up to 22.5 min of ECG recordings from patient-activated episodes and up to 27 min of ECG recordings from automatically detected arrhythmias. The devices also report daytime heart rate (HR) (08:00-20:00; referencing a 24 hour clock) and nighttime HR (0:00-04:00). For human patients, the ICM records cardiac information in response to both automatically detected arrhythmias and patient activation using a hand held device prescribed at the time of device implantation. Although designed for activation by a clinical patient during symptomatic episodes, the device can be activated by researchers and clinicians to record electrocardiograms during periods of interest. Arrhythmias that can be selected for automatic detection include: atrial tachyarrhythmias/atrial fibrillation (AT/AF), bradyarrhythmias (slow heart rates), asystole (long periods without a heart beat), and ventricular tachyarrhythmias (high heart rates).

We programmed devices after implantation in bears using a portable programmer, and used the same programmer to download data through the skin of bears visited at winter dens a year later. Devices were implanted in March 2009 and 2010, and follow-up visits were made the following December and March. In addition to continuously recording heart rates and activity, the devices were programmed to automatically detect and store the ECG for episodes in which: 1) a heart rate of at least 167 beats per minutes (bpm) was sustained for at least 16 beats ("tachycardia"), 2) a heart rate of less than 31 bpm was sustained for at least 4 beats ("bradycardia"), and 3) pauses of at least 4.5 seconds between consecutive heart beats ("asystole"). For purposes of data analyses, the period of winter inactivity (essentially the period of winter hibernation) was defined as the interval from when activity dropped below 1 hour/day in the fall to the time when activity of over 3 hours/day was sustained in the spring. Studies were conducted in conjunction with the Minnesota Department of Natural Resources and were approved by the University of Minnesota's Animal Care and Use Committee. All statistical analyses were performed using the non-parametric Mann-Whitney U-test. Normality was evaluated using a Shapiro-Wilk test. P-values less than or equal to 0.05 were considered significant.

## Results

Data were retrieved from 7 of 14 devices implanted in wild Minnesota black bears (*Ursus americanus*), and also from a bear that was kept in captivity over winter and released in spring. Annual HR and activity data were successfully retrieved from 6 bears and partial data sets were retrieved from 2 bears shot by hunters in the fall. The data from the other 7 devices were lost when bears were shot by hunters and not retrieved or when rejected by bears due to a foreign body response (as has been previously reported for other devices implanted in wild bears) [7,24]. The devices that remained implanted showed no evidence of inflammation or irritation. All sutures had been absorbed and the subcutaneous insertion sites (an incision of 1.5 cm) were no longer detectable. For the devices that were rejected, the implantation site was no longer detectable and could only be located via the patch of hair that had been shaved at the time of implant. The animals from which annual datasets were successfully collected included: two adult males, two females with cubs that denned with them during the subsequent winter as yearlings, a female with yearlings that became pregnant and gave birth to two cubs during the winter study period, and a female for which two consecutive years of data were obtained, who denned with yearlings during one winter and gave birth to cubs the second year.

Extremes in average daytime HR (08:00-20:00) ranged from 8 beats/minute (bpm) in the winter (during hibernation) to 135 bpm in the summer, with nighttime (0:00-04:00) averages of 7 to 139 bpm (i.e., similar ranges in daily averages for day and night). During the period of captivity, bear 5 had a maximum daytime average heart rate of 144 bpm and a maximum nighttime average heart rate of 150 bpm. These data were not included in the range reported above due to the unnatural conditions. The longest period of asystole confirmed with an ECG was 14.4 seconds in bear 3 during a period where the respiration rate was 1.53 breaths/minute (winter; equivalent heart rate of 4 bpm) and the maximum HR confirmed with an ECG was 214 bpm (summer). ICMs recorded an average of 25.0 ± 1.6 million heart beats/year for the bears (Range: 23.3 - 27.4 million beats/year). Activity sensors documented a minimum of 0 minutes (winter) to a maximum of 1084 minutes (summer) of activity over a 24 hour period. See Table 1.

**Table 1:** Summary of heart rate and activity data recorded over a 12 month period in wild black bears.

Animal ID	Sex	Implant Date (Den Visits)	Denning Situation	Heart Beats per Year#	Minimum Average Daily HR	Maximum Average Daily HR	Daytime HR Higher (p-value)	Minimum Average Nighttime HR	Maximum Average Nighttime HR	Nighttime HR Higher (p-value)	Annual Activity	Minimum Daily Activity	Maximum Daily Activity	Duration of Winter Inactivity@	Longest Sinus Pause Confirmed by ECG	Maximum HR Confirmed by ECG
Bear 1 (R2079)	F	9-Mar-09 (17-Dec-09, 04-Mar-10)	Denning with cubs 2008-9, and yearling in 2009-2010	27.5 × 10^6^	8 bpm (27-Dec)	120 bpm (14-May)	Feb (0.0099) Mar (0.023) Apr-May (< 0.0001) June (0.0058) July-Sep (< 0.0001)	11 bpm (6X: 02-Dec to 11-Feb)	139 bpm (15-May)	Oct (0.015)	139,384 min	0 min (5X: 17-Dec-09 to 3-Feb-10)	1084 min (2-Sep-09)	148 days	6.1 sec (04-Mar)	214 bpm (31-Oct)

Bear 2 (R2081)	F	9-Mar-09 (17-Dec-09, 04-Mar-10)	Denning with yearlings 2008-9, and birthing cubs in 2009-2010	23.5 × 10^6^	14 bpm (08-Nov, 11-Nov)	94 bpm (8-Sep, 9-Sep)	Feb (0.0084) Mar (0.0079) Apr (0.022) May-Sep (< 0.0001) Oct (0.041)	7 bpm (15-Nov)	87 bpm (19-Sep-09, 24-Sep-09)		106,320 min	0 min (4X: 13-Jan-10 to 1-Feb-10)	880 min (29-Jun-09)	189 days	5.3 sec (04-Mar)	200 bpm (18-Sep)

Bear 3 (2213)	F	8-Mar-09 (18-Dec-09, 05-Mar-10, 05-Mar-11)	Denning with cubs 2008-9, and yearlings in 2009-2010	24.9 × 10^6^	11 bpm (27-Nov)	114 bpm (07-Aug)	Apr (0.036) May (0.0007) June-July (< 0.0001) Aug (0.01)	11 bpm (26-Nov, 27-Nov, 8-Dec)	124 bpm (3-Sep-09)		108,900 min	0 min (34X: 26-Oct to 26-Feb)	1046 min (6-Aug-09)	195 days	14.4 sec (18-Dec)	182 bpm (7-Oct)

Bear 3 (2213) Year 2	F	8-Mar-09 (18-Dec-09, 05-Mar-10, 05-Mar-11)	Denning with yearlings 2009-2010 and birthing cubs in 2010-2011	25.9 × 10^6^	12 bpm (26-Oct)	109 bpm (26-Jun)	Apr (0.099) May (0.0005) Jun-July (< 0.0001)	7 bpm (7-Nov)	126 bpm (3-Sep)	Sep (0.0043)	101,437 min	0 min (5X: 7-Jan to 13 Jan)	1051 min (26-Jun)	194 days	N/A$	N/A$

Bear 4 (4041)	M	2-Mar-09 (22-Dec-09, 07-Mar-10)	Denning alone	23.4 × 10^6^	12 bpm (4X: 20-22-Dec, 16-Feb)	105 bpm (19-Aug)	May (0.030) June (0.0002) July (0.019)	12 bpm (13X: 24-Nov to 16-Feb)	113 bpm (22-Aug)	Sep (< 0.0001) Oct (0.0051)	31,346 min%	20 min% (1-Apr)	1028 min% (8-Jun)	N/A%	10.1 sec (07-Mar)	182 bpm (15-Oct)

Bear 5& (2123)	M	1-Mar-10	Denning in rescue facility. Released 7-May.	N/A	35 bpm& (19-Mar)	144 bpm& (29-Apr)	Mar (0.0042) May (0.0075) June-Aug (< 0.0001) Sep (0.0029)	32 bpm& (11-Mar)	150 bpm& (6-May)		N/A	4 min& (11-Mar)	1030 min& (19-Aug)	N/A&	N/A$	251 bpm (2-Sep)

Bear 6& (2802)	F	7-Mar-10	Yearling that denned with mother	N/A	35 bpm& (10-Mar)	135 bpm& (9-Jul)	Mar (0.036) Apr (0.0003) May-Aug (< 0.0001)	32 bpm& (10-Mar, 11-Mar)	121 bpm& (21-Aug)		N/A	8 min& (7-Mar)	973 min& (23-Aug)	N/A&	N/A$	N/A$

Bear 7 (4055)	M	9-Mar-10 (22-Dec-10)	Denning alone	N/A	16 bpm (3X: 11-Dec, 16-Dec, 20-Dec)	128 bpm (21-May)	Mar (< 0.0001) Nov (< 0.0001) Dec (0.020)	15 bpm (8X: 19-Nov to 21-Dec)	119 bpm (23-Dec)	May (0.0002) July (0.047) Aug-Sep (< 0.0001)	100,512 min*	0 min* (10-Dec)	1034 min* (9-Jun)	176 days	N/A$	N/A$

Bear 8 (4021)	F	9-Mar-10 (09-Mar-11)	Denning with cubs in 2009-2010 and yearlings in 2010-2011	25.3 × 10^6^	15 bpm (21-Nov, 5-Dec)	101 bpm (19-Jul)	Mar (0.020) Apr-July (< 0.0001) Aug (0.0021)	15 bpm (14-Feb, 6-Mar)	97 bpm (1-Sep)	Sep (< 0.0001)	112,351 min	0 min (51X: 14-Nov to 7-Mar)	980 min (19-Jul)	157 days	N/A$	N/A$

% Incomplete data set for activity (4-Mar to 9-Jun)

* Data set is less than 12 months (9-Mar to 22-Dec)

# Calculated by extrapolating daytime and nighttime heart rates to a 24 hour period.

& Legally shot by hunters in the fall. Incomplete annual data for this parameter.

$ No ECG available to confirm this parameter.

@ The duration of winter inactivity was defined as starting when the activity dropped to below 60 minutes/day in the fall until it consistently exceeded 180 minutes/day in the spring.

(min = minutes; bpm = beats per minute; HR = heart rate; 6X = 6 occurrences of this data value; N/A = not applicable to this parameter)

**Figure 1:**
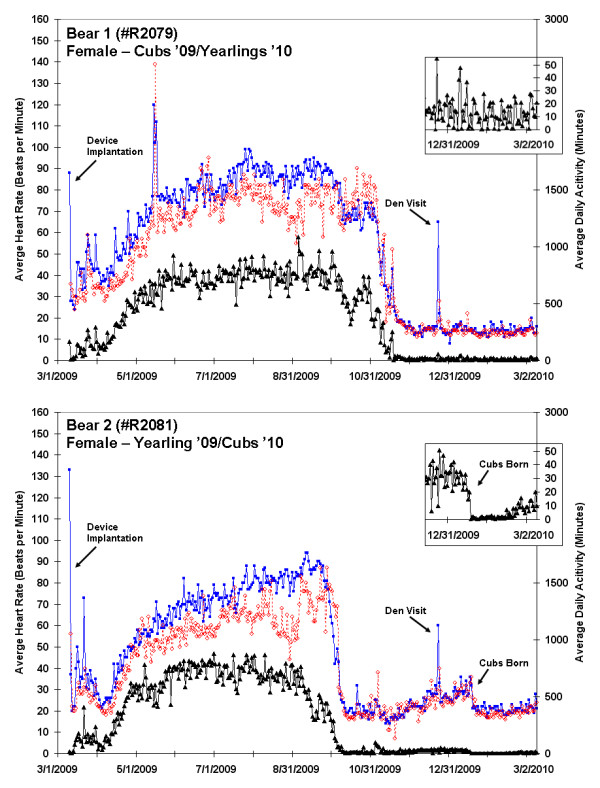
**Twelve month heart rate and activity data for Bear 1 (female denning with cubs in 2009 and yearlings in 2010) and Bear 2 (female denning with yearlings in 2009 and giving birth to cubs in 2010)**. Average daytime heart rate (blue; 08:00-20:00), average nightly heart rate (red; 0:00-04:00 AM), and daily activity (black line) are shown. Initiation of denning, elevated heart rates following device implantation and during den visit in mid-December, and a shift towards nocturnal behavior in the fall are evident. An expanded plot of the daily activity during the second winter is included. Bear 2 gave birth to cubs in January 2010 with a reduction in both activity and heart rate seen in subsequent days.

**Figure 2:**
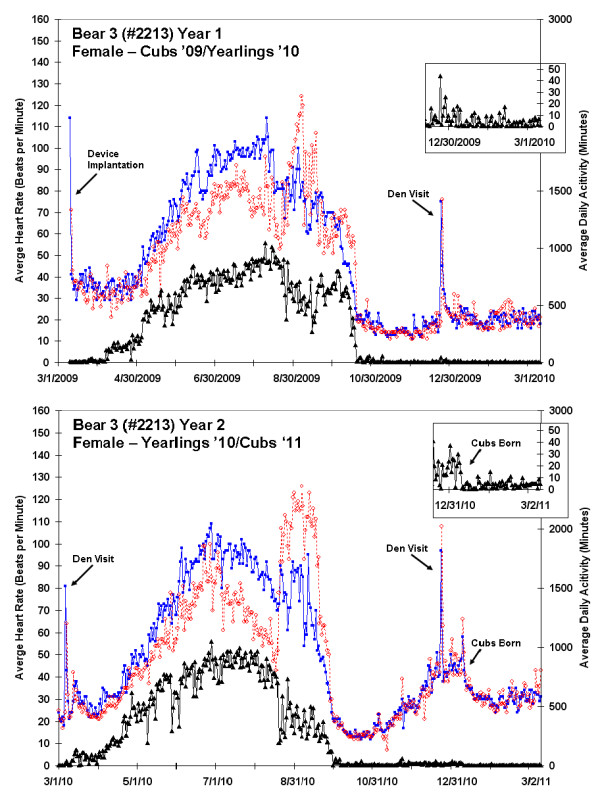
**Twelve month heart rate and activity data for two consecutive years from Bear 3 (#2213; female denning with cubs in 2009 and yearlings in 2010, and giving birth to cubs in 2011)**. Average daytime heart rate (blue; 08:00-20:00), average nightly heart rate (red; 0:00-04:00 AM), and daily activity (black line) are shown. Initiation of denning, elevated heart rates following device implantation and during den visit in mid-December, and a shift towards nocturnal behavior in the fall are evident. An expanded plot of the daily activity during the same portion of both winters is included. Bear 3 gave birth in January 2011 with a reduction in both activity and heart rate seen in subsequent days.

**Figure 3:**
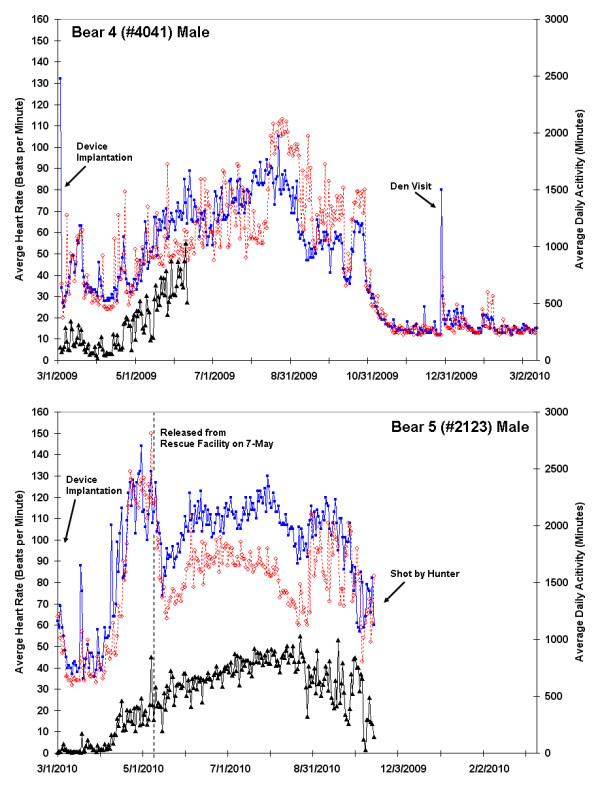
**Twelve month heart rate and activity data for Bear 4 (#R4041; male) and Bear 5 (#2123; male shot by hunter)**. Average daytime heart rate (blue; 08:00-20:00), average nightly heart rate (red; 0:00-04:00 AM), and daily activity (black line) are shown. Initiation of denning, elevated heart rates following device implantation and during den visit in mid-December, and a shift towards nocturnal behavior in the fall are evident. The ICM in bear 4 ceased reporting activity data in early June during a period associated with an elevated heart rate which is the height of the breeding season. The cause of the data loss is not yet known since the device remains implanted, but the activity circuitry may have been damaged. Bear 5 was legally shot and killed by a hunter during the study period, resulting in a cessation of data collection. This bear was held in a wildlife recovery center until late spring, resulting  in an a typical increase in heart rate and activity during the month of May.

Trends and levels of HR and activity (time active per day) were similar for the six animals with complete data sets (representative examples shown in Figures 1, 2 and 3). Both HR and activity increased through the spring for at least a month following emergence from hibernation in early April. Activity then reached a generally steady state for several months, whereas HRs continued to increase over the summer months. During the spring and summer months the population of bears was more diurnal, as indicated by the statistically higher daytime HRs for the months of March-August, and generally more nocturnal in the months of September and October. Although nocturnal activity did not achieve significance for the population in any single month, nighttime HR was significantly higher at least once in 5 of 6 bears in September and/or October (excludes bears shot by hunters in the fall; detailed in Table 1).

A sharp decline in both HR and activity occurred in September and October, with the first day of denning evident from a dramatic drop in activity (Figures 1, 2 and 3). The duration of winter inactivity (including 2 winters for bear 3) was 176 ± 20 days (range: 148 to 195 days) with only 24 ± 6% of heart beats occurring during this period. A sharp increase in heart rate was evident in late December for all bears, corresponding to the winter den visit by the research team; this elevated rate was sustained for 1-2 days after our disturbance. During our March visit to the den of the female with newborn cubs (bear 2), there were fresh wolf tracks near the den entrance. This encounter resulted in only a subtle increase in activity and heart rate, as there were no large spikes in the record. A sharp cessation of activity in mid-January preceded by a period of elevated heart rate is seen in the expanded plot for bear 2 (Figure 1) and in the second year for bear 3, corresponding to the birth of cubs. The mother may have remained in a more stationary position immediately after birthing so as not to crush the altricial cubs, which stay warm underneath her, and to provide them constant access to milk. Changes in mid-winter activity were not observed for the females with yearlings (which do not nurse during winter). The ICM in bear 4 (an adult male) ceased collecting activity data in early June during the height of the breeding season. The cause of the data loss is not yet known because the device remains implanted, but we suspect that the activity circuitry may have been damaged since cessation of data collection correlated to a period of high heart rate and activity. The device lies just under the skin, so is potentially vulnerable to damage from high impact.

The trend data for one of two bears legally shot by a hunter is shown in the lower panel of Figure 3 (bear 5). The general trends in heart rate and activity are similar to bears 1-4 with the exception of the late winter/springtime data during which time this bear was housed outdoors in a wildlife rescue facility. Although the activity levels (physical movement) of bear 5 were very limited and appeared to be similar to the other bears in late hibernation, the bear's heart rate was substantially elevated until it was released into the wild (May 7). The data from bear 5 were included here because they demonstrate the physiological reaction to the hunt and also highlight the disparity in heart rates between captive and free-ranging bears.

**Figure 4:**
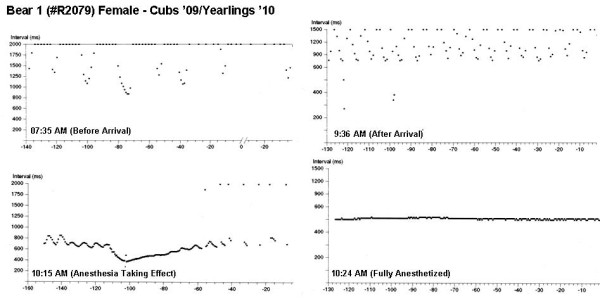
**Heart rate changes in a wild bear during a visit to the den site**. Sequences automatically recorded on 17-Dec-09 during a visit to the den of bear 1. Panels show heart rate trends: prior to arrival at the den (slow heart rate with RSA), after arrival (team arrived at 9:30 AM; respiration rate noticeably elevated), while the anesthetic was taking effect (increase in heart rate and attenuation of RSA), and of the fully anesthetized animal (no RSA and steady rate of 120 bpm). The downward excursions in the plot correspond to the respiratory cycling, and are a result of a pronounced respiratory sinus arrhythmia.

A unique feature of the ICM is the automatic generation of HR plots and the recording of ECGs during triggered events. The plots compare the interval between consecutive heart beats in milliseconds on the abscissa (1000 msec interval corresponding to a HR of 60 bpm) to time in seconds on the ordinate axis. The plots were truncated by the ICM at an interval of 1500 msec since such slow rates were not anticipated in human clinical use. Examples of HR plots recording during the team's approach to the den site of bear 1 are shown in Figure 4. A slow HR and respiration rate, with a pronounced respiratory sinus arrhythmia (RSA) was present at 07:35 prior to the team's arrival. Upon our approach to the den at 09:36 both the HR and respiration rate accelerated, even though we made a concerted attempt to be very quiet. The accelerated HR and respiration continued, with a dampening of the RSA following administration of the immobilizing drugs but prior to the bear being fully anesthetized. A stable rate of approximately 120 bpm with no RSA occurred under the full effect of the anesthetic agent. This series was typical of the bears studied and demonstrates the alertness of the bear while in a state of hibernation to potential dangers outside the den. Such physiological responses were not always apparent through simply observing the behavior and activity of the bear in the den.

**Figure 5:**
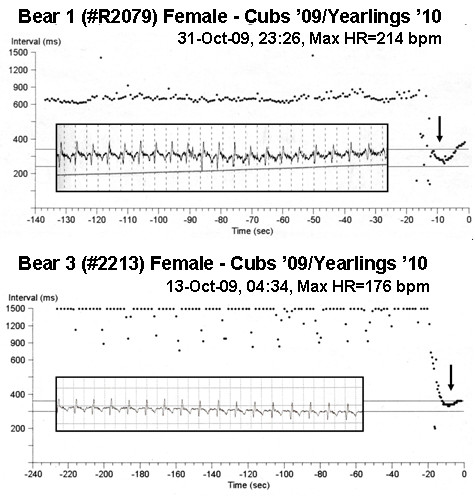
**Elevated heart rates recorded from two free-ranging wild black bears**. The top panel demonstrates heart rate acceleration from a steady rate of approximately 85 bpm to 214 bpm. The bottom panels shows an acceleration during a period with a pronounced respiratory sinus arrhythmia (RSA). Note that the respiration rate is readily apparent in the lower panel from the RSA (4 breaths/minute; 14 breaths taken over a period of 220 seconds). It is postulated that this elevation in heart rate is due to a deepened breath. A sample of the ECG during the maximum recorded heart rate is inset in each panel (each interval on the ECG strip is equal to 0.2 seconds). The arrow indicates the approximate center point of the ECG strip.

Extremes in HR are demonstrated in Figures 5. Examples are shown for two bears that survived the entire year, including both the HR trend plot and an embedded ECG correlating to the period of maximum HR. A heart rate of 214 bpm was confirmed from the ECG trace for bear 1. The upper panel in the figure demonstrates a HR acceleration from a stable heart rate of approximately 85 bpm to a rate of over 200 bpm. The lower panel demonstrates a HR acceleration from a period with a pronounced RSA. The acceleration to a rate of 176 bpm appears to be associated with an exaggerated respiratory cycle. The presence of the RSA is evidenced by the oscillations in the HR and can be confirmed by modulations in the ECG amplitudes detected by the ICM. This amplitude modulation is a result of variations in intra-body impedances during chest expansions and lung inflations [25,26]. From analysis of these oscillations on the embedded ECG on the lower panel of Figure 5, the respiration rate of bear 3 was found to average 4 breaths/minute. HR cycle lengths of less than 200 msec were recorded in other episode plots (300 bpm equivalent) but were always associated with oversensing of T-waves by the ICM. Only the maximum and minimum values that could be confirmed with ECGs were reported to eliminate the possibility of erroneous rhythm interpretation by the device.

**Figure 6:**
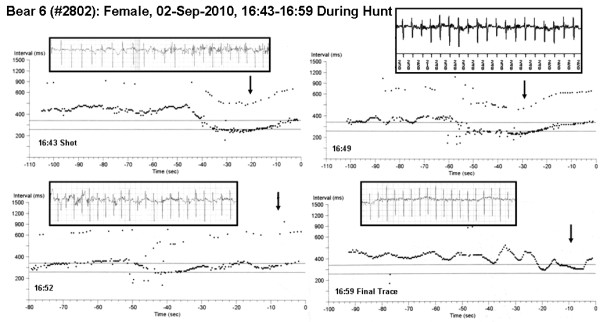
**ECG trends from a hunted bear**. Sequences automatically recorded from 16:43 - 16:59 on 02-Sep-10 during the time period when bear 6 was legally shot and killed by a hunter. This device was returned to the research team by the hunter. Panels show heart rate trends at the time the bear was shot (upper left) and for the 15 minutes following. The panel at the lower right contains the final heart rate data from this bear. Electromyograms are apparent in the upper left and lower left panels. During the time period shown in the upper right panel (16:49) the heart rate averaged 251 bpm (the numbers at the bottom of the inset ECG trace are the intervals between consecutive heart beats in milliseconds: for example, 230 ms corresponds to a heart rate of 261 bpm).

The highest heart rate documented during this study was from an animal that was legally shot during the fall hunting season (bear 5; Figure 6). Although this bear was not detected by the hunter's trail camera at his bait site until just before it was shot, the bear's HR exceeded 200 bpm for 17 episodes in the 3 hours prior to being shot, suggesting that the bear was in the area of the hunter's bait and sensed danger. The final recordings from this bear included an interval with an average heart rate of 251 bpm, with a minimum interval between consecutive heart beats of 210 milliseconds (corresponds to an instantaneous heart rate of 285 bpm; see the upper right panel in Figure 6). The final recordings for the second bear shot by a hunter (bear 6) indicated an average HR of 200 bpm sustained for 64 seconds with a peak of 207 bpm.

**Figure 7:**
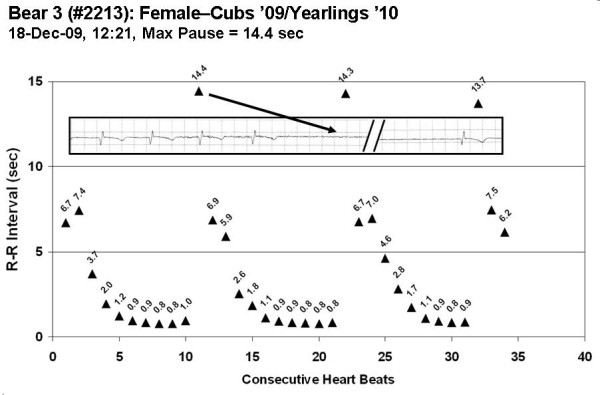
**Heart rate intervals for three consecutive respiratory cycles from a free-ranging wild black bear**. The heart rate intervals for 34 consecutive beats were recorded on 18-Dec-09 at 12:20 PM for bear 3 (#2213). The ECG inset in the figure corresponds to the first respiratory sequence with the location of the 14.4 second pause indicated by the arrow. This pause is equivalent to a transient heart rate of 4.2 bpm. The average respiration rate during this period was 1.53 breaths/minute (3 breaths occurring over an interval of 117.6 seconds). Each interval on the embedded ECG strip is equal to 0.2 seconds.

The longest period of asystole confirmed with an ECG trace is plotted in Figure 7. Bear 3 had three consecutive respiratory cycles documented with ECG recordings, and showed sinus pauses of 14.4, 14.3, and 13.7 seconds. The average respiratory rate during this period was 1.53 breaths/minute (3 breaths in 117.6 seconds). The morphology of the ECG was similar before and after the pause, indicating that it is a natural heart beat emanating from the sinoatrial node. All animals filled the available device memory for asystolic events, with 65,565 sinus pauses of at least 4.5 seconds documented.

**Figure 8:**
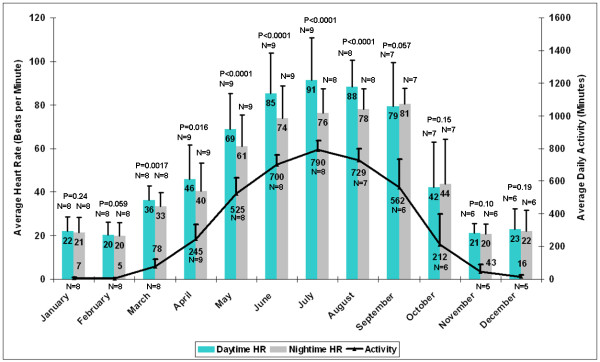
**Annual trends in heart rate and activity in free-ranging wild black bears**. The average daytime heart rate, nighttime heart rate, and daily activity are plotted (two years are included for bear 3). The error bar indicates one standard deviation. The sample size available for each data point is indicated. Although seasonal changes in heart rate and activity were significant, there was no statistical difference found between the daytime and nighttime heart rate for any one month interval.

The cumulative heart rate and activity results are summarized in Figure 8. All data were included in this analysis, with the exception of the data related to the period of captivity for bear 5. As expected, a dramatic decrease in heart rate and activity are evident during the winter months. Although a statistically significant shift to nocturnal behavior in the fall was seen when comparing the average daytime and nighttime heart rates for each month for individual bears, the difference was not significant for any single month when viewed across the population.

## Discussion

This is the first investigation of continuous annual heart rhythms and associated body acceleration activities recorded from bears in the wild. In addition, we believe this to be the first recording of physiological parameters from an animal hunted in the wild. The ICMs yielded 24 hour data throughout a 12 month period, providing contrasts between periods of hibernation and non-denning activity, and a record of the transitions between the two. The devices provided valuable insights into the otherwise non-obvious influence on the bear's physiology and behavior during data collection activities, as well as behaviors associated with birthing, cub-rearing, hunting, and natural seasonal variations. Although the general trends seen in heart rhythms during summer and winter data collection intervals were similar to those reported in previous studies, we documented natural extremes in both high and low heart rates that exceeded any previously recorded in either wild or captive bears [7,19-22]. Our observation of bears becoming more nocturnal in fall is consistent with activity data collected from radio-collared black bears in Minnesota and elsewhere, and may be related to a change in the types of food eaten, thermoregulatory responses to increased body mass, and/or avoidance of hunters [27,28].

## Conclusions

The effects of environmental changes on animals like bears are often assessed by investigating movements, habitat use, and stress hormones, but these techniques have a number of limitations [29-33]. We suggest that implantable heart and activity monitors that are made for use in humans, and thus seeing rapid technological improvement, are readily adapted for monitoring behavioral and physiological changes in wild animals. The device used in this study enabled the collection of cumulative annual activity and number of annual heart beats, allowing for the longitudinal assessments of the physiological stress imposed by such factors as human encroachment and climate change on wild cohorts. To date, we have not yet fully exploited the opportunities offered by this technique. A future step might entail a comparison of heart rates with ambient temperature, which varied from -30 to 42°C during this study. Additionally, the study bears all had radio-collars with GPS units that stored locational data that can be matched to habitat. Thus, we expect to be able to ultimately investigate heart rhythms and activity patterns as bears moved from dense forest to open fields, crossed roads, came near houses, and fed in agricultural fields. Our study site is at the extreme western edge of the bear range for the eastern United States, and contains a patchwork of small woodlots interspersed with agriculture. Much of the day-to-day variation in average heart rates exhibited over the course of the active season (Figures 1, 2 and 3) was likely due to bears moving across this patchwork of habitats, probing the limits of their range, and at some level, interacting with anthropogenic aspects of their environment. Novel insights into how they react biologically to this environment are likely to be gained through a complete record of their heart beats. We thus suggest that ICMs may be a useful addition to the burgeoning field of conservation physiology [34]. New devices, applications, and procedures for heart rate monitoring will continue to advance the growing body of literature investigating effects of human activities and other environmental stressors on wildlife [35-40].

## Competing interests

TGL is an employee of Medtronic, Inc. PAI is a consultant to Medtronic.

## Authors' contributions

TGL and PAI planned the experiments, TGL, PAI, and DLG conducted the fieldwork. TGL led in manuscript preparation, but all authors contributed to the ideas and writing. All authors read and approved the final manuscript.

## Supplementary Material

Additional file 1 **Hibernating female bear video 1**. Hibernating female bear with cubs prior to tranquilization March 2008. Click here for file

Additional file 2 **Hibernating female bear video 2**. Bear 3 prior to tranquilization on 18-Dec-2009. Click here for file

Additional file 3 **Hibernating female bear video 3**. Bear 3 prior to tranquilization in March 2009 with audible cub sounds. Click here for file

Additional file 4 **Hibernating female bear video 4**. Female bear with cubs March 2009 with audible cub sounds. Click here for file
